# Danhong Injection (a Traditional Chinese Patent Medicine) for Acute Myocardial Infarction: A Systematic Review and Meta-Analysis

**DOI:** 10.1155/2015/646530

**Published:** 2015-09-14

**Authors:** Pengda Liao, Lei Wang, Liheng Guo, Ruixiang Zeng, Juming Huang, Minzhou Zhang

**Affiliations:** ^1^Guangdong Provincial Hospital of Chinese Medicine, Guangzhou 510120, China; ^2^The 2nd Clinical College of Guangzhou University of Chinese Medicine, Guangzhou 510405, China

## Abstract

*Objective*. We aimed to systematically assess the efficacy and safety of Danhong injection (DHI) for acute myocardial infarction (AMI) patients. *Methods*. We searched several electrical databases and hand searched several Chinese medical journals. Randomized controlled trials (RCTs) comparing DHI plus conventional western medicine with conventional western medicine plus placebo and RCTs comparing DHI plus conventional western medicine with conventional western medicine were retrieved. Study screening, data extraction, quality assessment, and data analysis were conducted in accordance with the Cochrane standards. *Results*. 13 RCTs enrolling 979 patients were included. Danhong injection could significantly reduce the risk of mortality, recurrent angina, arrhythmia, and heart failure. In addition, DHI was associated with improvement of left ventricular ejection fraction (LVEF) and reperfusion. No significant difference of DHI was found on recurrent acute myocardial infarction. However, the safety of DHI remained unknown for limited data. *Conclusion*. DHI might be a potentially efficacious treatment for AMI patients. Nevertheless, the safety of DHI remained uncertain for limited information. Due to the fact that the overall quality of all included studies is generally low, more high quality RCTs are expected to validate the efficacy and safety of DHI for AMI patients.

## 1. Introduction

Acute myocardial infarction (AMI), a lethal type of coronary heart disease (CHD), is one of the major causes of death in the world [1]. AMI occurs when coronary artery is occluded, usually on the basis of rupture, thrombosis, or erosion of the coronary atherosclerotic plaque, leading to acute inadequate blood flow and oxygen supply to heart muscle [2]. AMI is the most common cause of morbidity among ischemic heart diseases and is the leading death cause in the western world [3]. Even though the application of revascularization, including thrombolytic, percutaneous coronary intervention (PCI) and coronary artery bypass grafting (CABG), has successfully reduced AMI patients' mortality, they are still facing certain risk of in-hospital death [4]. Being in a dilemma, revascularization is also associated with intractable complications, for example, no-reflow phenomenon after PCI, intrastent thrombosis, and ischemia-reperfusion injury [5]. Since the frequent and successful use of Traditional Chinese Medicine (TCM) in the prevention and treatment for CHD, the effects of TCM for CHD have aroused increasing attention [6–8].

Danhong injection (DHI), a Chinese patent compound injection, is widely used in the treatment for several diseases, including AMI. DHI consists of two components, roots of danshen (*Radix Salvia Miltiorrhizae*) and flower of honghua (*Flos Carthami Tinctorii*) [9]. Previous studies have shown that danshen, the main constituent of DHI, could be a vasodilator, lowering the vascular resistance and blood viscosity so as to protect myocardium [10–12]. Moreover, honghua has been demonstrated to possess multiple pharmacological characteristics, including vasodilation, antioxidation, calcium antagonism, and oxygen-free-radical scavenging [13–15]. Recent pharmacological researches have also shown that DHI has a positive effect in inhibiting the aggregation of platelet, improving AMI patient's hemodynamic status and endothelial function [16–18]. Meanwhile, a large number of clinical trials also revealed the positive efficacy of DHI for AMI patients. Thus, DHI might be a potentially effective medicine for AMI. However, the efficacy and safety of DHI for AMI have not yet been systematically assessed. So our study aimed to assess the efficacy and safety of DHI plus the conventional treatment for AMI patients.

## 2. Methods

### 2.1. Inclusion and Exclusion Criteria

All randomized controlled trials (RCTs) comparing DHI plus conventional western medicine with conventional western medicine plus placebo and RCTs comparing Danhong injection plus conventional western medicine with conventional western medicine were included, regardless of their publication status, population characteristics, or languages. Patients meeting any one of the current or previous AMI diagnostic criteria [19–23] were included, with no limitation to their gender, age, religion, or ethnic origin. Trials that did not provide description of diagnostic criteria but declared patients with definite AMI were also considered. Primary outcomes included mortality, recurrent AMI, and reperfusion rate. Secondary outcomes included heart failure, arrhythmia, left ventricular ejection fraction (LVEF), recurrent angina, and adverse events. Quasi-randomized controlled trials, animal trials, pharmacological trials, and duplicated trials were excluded. In addition, trials whose difference in sample size between DHI group and control group was greater than 50% were also excluded so as to ensure the precision of the study.

### 2.2. Database and Search Strategies

We extensively searched published and unpublished RCTs in the following databases: PubMed, The Cochrane Library (Issue 12, 2014), Embase, Chinese Biomedical Database (CBM), Chinese VIP Information (VIP), China National Knowledge Infrastructure (CNKI), and Wanfang Databases, with search terms adjusted to each database as followed: “Danhong injection,” “acute myocardial infarction,” “coronary artery disease,” “acute coronary syndrome.” We searched ongoing registered clinical trials on the website of WHO International Clinical Trial Registry Platform (http://apps.who.int/trialsearch/) and international clinical trial registry by US national institutes of health (http://www.clinicaltrials.gov/). Our entire search was ended on December 28, 2014. Besides, bibliographies of the included studies were searched to avoid missing relevant articles.

### 2.3. Study Selection and Data Extraction

Two authors (Juming Huang and Ruixiang Zeng) independently scanned the search results by titles and abstracts and selected potentially relevant RCTs. Full texts of potentially relevant articles were retrieved. Based on the inclusion and exclusion criteria, articles were further identified. Data of included studies was extracted and filled into a prespecified electronic form by two authors, Juming Huang and Ruixiang Zeng independently. The extracted data included authors, title, and year of publication, sample size, age and sex of the participants, information of methodological quality, details of the treatment for both groups, outcomes, and adverse effects for each study. Disagreement (if any) was solved by discussions with a third author (Lei Wang).

### 2.4. Risk of Bias Assessment

In accordance with the Cochrane Collaboration's risk of bias assessment tool [37], two authors (Juming Huang and Ruixiang Zeng) independently evaluated the methodological quality of all included studies via the following aspects: random sequence generation (selection bias), allocation concealment (selection bias), blinding of participants and personnel (performance bias), blinding of outcome assessment (detection bias), incomplete outcome data (attrition bias), selective reporting (reporting bias), and other biases. For each aspect, a low risk was considered when we judged a “Yes,” conversely, a “No” for a high risk, and otherwise for an unclear risk. Efforts were made to obtain missing information from the original authors whenever possible. Discrepancies were resolved by consultation with a third author (Lei Wang).

### 2.5. Data Analysis

Revman 5.3 software from Cochrane Collaboration was applied for data analyses. Dichotomous data were presented as risk ratio (RR) and continuous outcomes as mean difference (MD), both with 95% confidence interval (CI). We always performed intention-to-treat (ITT) analysis to analyze data whenever possible. Fixed effects model was applied to analyze data if there was low heterogeneity (*I* ^2^ ≤ 50%); random effects model was used if there was high heterogeneity (50% < *I* ^2^ < 75%). Data were not pooled if there was significant heterogeneity (*I* ^2^ ≥ 75%) [37], in which case we explored potential causes of heterogeneity by conducting subgroup analyses based on the characteristics of intervention (dosage, duration), the types of conventional therapy (PCI versus thrombolysis), and the methodological quality. Sensitivity analysis was applied on low methodological quality studies in order to investigate whether including such studies would alter the results. Publication biases were explored by funnel plot analysis if the number of included studies of any outcomes was greater than ten [37].

## 3. Results

### 3.1. Search Flow

**Figure 1:**
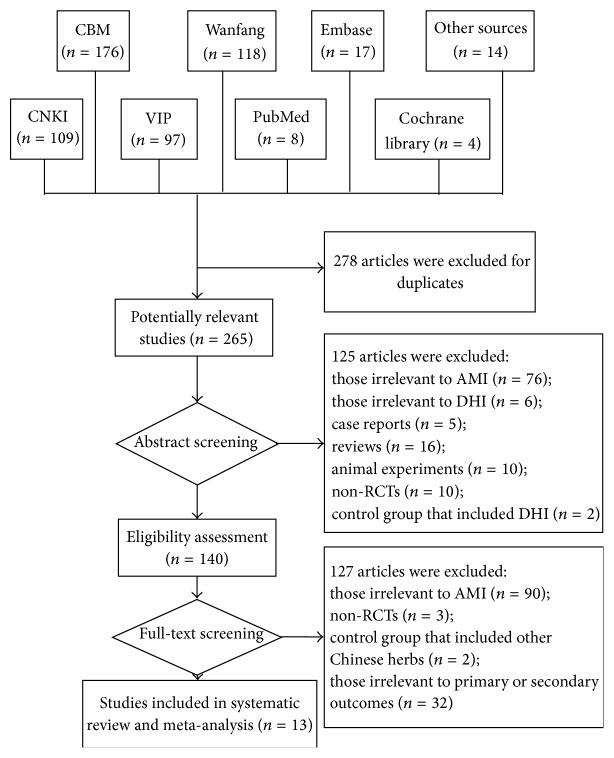
Flowchart of study search and identification. Notes: CNKI: China National Knowledge Infrastructure; CBM: Chinese Biomedical Database; VIP: Chinese VIP Information; AMI: acute myocardial infarction; DHI: Danhong injection.

A flow diagram demonstrated the search process and study selection (Figure 1). According to the preset search strategy, a total of 543 studies were found, of which 278 were excluded for duplicates. After reading the titles and abstracts, we excluded 125 articles for different reasons. 140 potentially eligible articles were retrieved for further assessment, of which 127 were excluded for the following reasons: being irrelevant to myocardial infarction (*n* = 90), being irrelevant to primary or secondary outcomes (*n* = 32), non-RCTs (*n* = 3), and control group that contained other therapies of Chinese medicine (*n* = 2). Therefore, 13 studies [24–36] were included. We also found one ongoing trail via US national institutes of health. This trial was not included in our review because it was recruiting participants.

### 3.2. Description of Included Studies

The characteristics of the 13 included studies [24–36] are summarized in Table 1. All these studies were conducted in China and published in Chinese. Among 13 included studies, two were postgraduate dissertation [33, 34], one was conference proceedings [36], and others were journal articles published from 2008 to 2013. The sample size in each of the included studies ranged from 40 to 134, with a total of 979 AMI patients in 13 included studies. Male (601) participants were more than female (378) participants. The age of participants was widely distributed, ranging from 36 to 85 years old.

Participants were diagnosed with AMI via different criteria: the CSCCMA diagnostic criteria were used in five studies [30, 32–34, 36]; the WHO diagnostic criteria were used in one study [29]; ACC/AHA diagnostic criteria were used in one study [24] and six studies [25–28, 31, 35] that failed to give a detailed description of their diagnostic criteria but mentioned “participants with AMI were included.” STEMI participants were included in two studies [24, 29], NSTEMI participants were included in one study [34], and participants that were included in the other studies were unclear about the types of AMI [25–28, 30–34, 36]. Detailed baseline information was available in six studies [26, 27, 29, 30, 32, 35]. In each of the included studies, baseline difference between experiment group and control group revealed no statistical significance.

All participants in the intervention groups received DHI plus conventional therapy while control groups received conventional treatment. Twelve studies [24–34, 36] specified the doses of DHI they used, ranging from 20 mL to 40 mL, while one study did not introduce the specific doses [35]. Eleven studies [24–29, 31–34, 36] reported the duration of treatment (from 7 days to 4 weeks) and length of follow-up (from 1 week to 3 months), while the other two studies did not.

Mortality was reported in six studies [25, 27, 30–32, 35]. Recurrent AMI was reported in two studies [25, 31]. Seven studies provided information of reperfusion [26, 27, 30–32, 35, 36]. Six studies provided the number of patients with recurrent angina [24, 25, 29, 32, 33, 36]. Arrhythmia was reported in eleven studies [25, 26, 28–36]. Eight studies offered information of participants who suffered from heart failure [25, 26, 29–33, 35]. Five studies reported the outcomes of LVEF [26, 28, 31, 34, 36]. As for adverse events, five studies [25, 27, 30, 32, 33] provided numerical cases on bleeding events and one study [34] gave a narrative introduction of liver and kidney functions.

### 3.3. Methodological Quality of Included Trials

**Figure 2:**
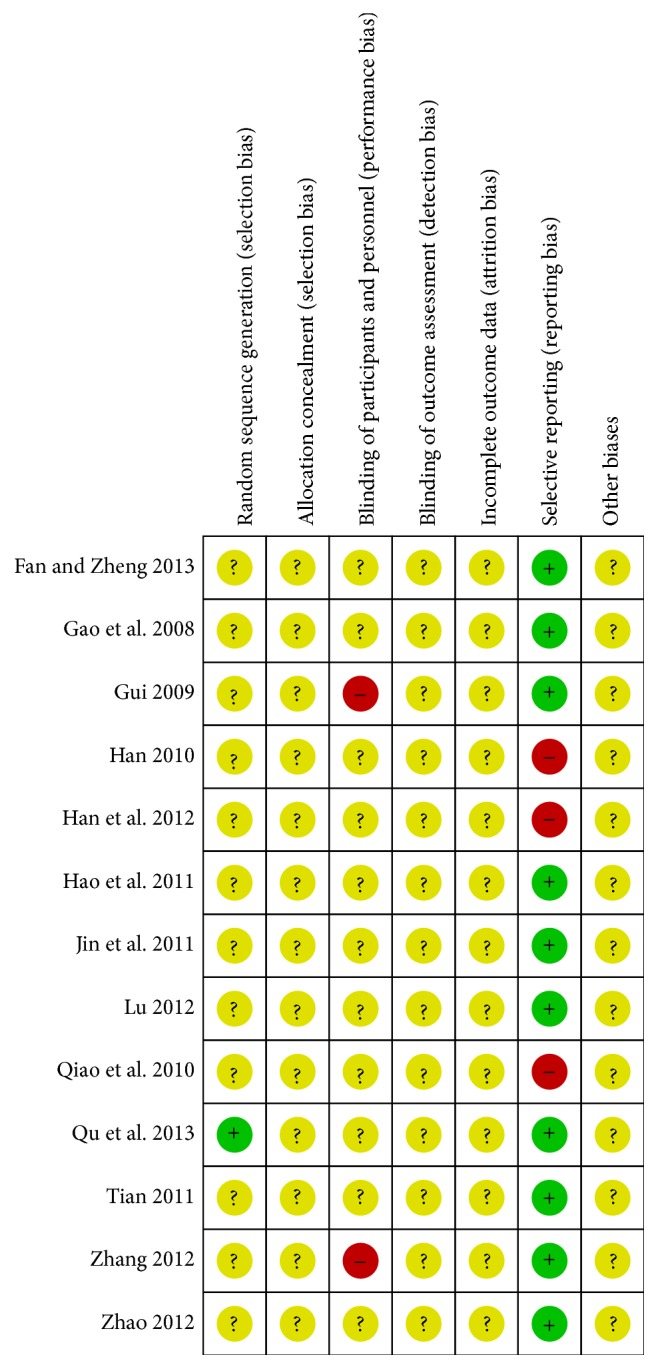
Risk of bias summary: review authors' judgment on each risk of bias item for each included study.

Risk of bias assessment of all included studies is presented in Figure 2. According to Cochrane Collaboration criteria, all of the thirteen included trials were evaluated as low methodological quality. Only one [36] of the thirteen included studies reported that random sequence was generated from a random number table, while other studies failed to account for the random sequence generation. None of the included studies described the allocation concealment. Two trials [25, 33] mentioned that they were single-blinded and the remaining trials did not report blinding of participants or personnel. Blinding of outcome assessment was not mentioned in any studies. Neither withdrawals nor losses to follow-up were reported in the included studies. Three trials [26, 27, 32] were considered to be associated with selective outcome reporting, because some outcomes were omitted or incomplete. No study mentioned prior sample size estimation or ITT analysis for any outcome. We tried every effort to contact authors by telephone, email, and other ways for further information about the trials. No other information was obtained.

### 3.4. Effect of the Interventions

#### 3.4.1. Mortality

**Figure 3:**
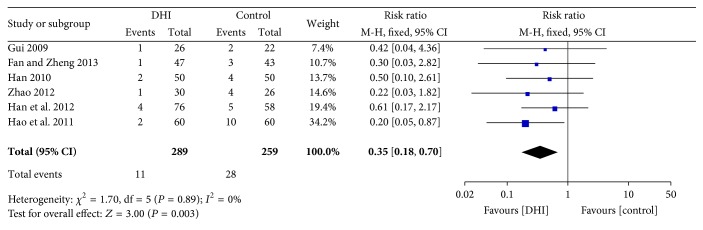
Forrest plot of mortality.

Six studies [25, 27, 30–32, 35] reported mortality. Meta-analysis showed statistically significant difference in the risk of mortality between conventional treatment plus DHI and conventional treatment (RR: 0.35; 95% CI 0.18 to 0.70; *n* = 548; *I* ^2^ = 0%) (Figure 3).

#### 3.4.2. Recurrent AMI

**Figure 4:**
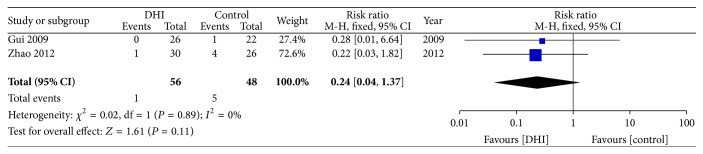
Forrest plot of recurrent acute myocardial infarction (AMI).

Two studies [25, 31] reported recurrent AMI. Meta-analysis showed no statistically significant difference in the risk of recurrent AMI between conventional treatment plus DHI and conventional treatment (RR: 0.24; 95% CI 0.04 to 1.37; *n* = 104; *I* ^2^ = 0%) (Figure 4).

#### 3.4.3. Reperfusion

**Figure 5:**
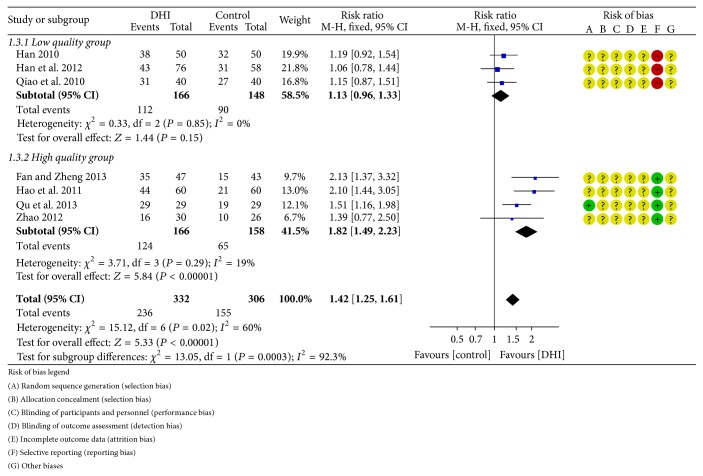
Forrest plot of reperfusion rate.

Reperfusion was reported in seven studies [26, 27, 30–32, 35, 36]. Meta-analysis (random effect model) revealed that conventional treatment plus DHI was associated with a statistically significant increase in reperfusion compared with conventional treatment (RR: 1.41; 95% CI 1.15 to 1.72; *n* = 638; *I* ^2^ = 60%). Since significant heterogeneity was observed, we rechecked these studies carefully and found out the difference of methodological quality among seven studies. Three trials [26, 27, 32] were assessed as low quality in selective outcome reporting, while the rest of the trials were of high quality in this aspect. Therefore, a subgroup analysis was conducted according to the methodological quality among seven studies. In the low quality subgroup [26, 27, 32], meta-analysis result (fixed effect model) changed to be statistically insignificant with no heterogeneity (RR: 1.13; 95% CI 0.96 to 1.33; *n* = 314; three studies; *I* ^2^ = 0%). In the high quality subgroup [30, 31, 35, 36], meta-analysis result (fixed effect model) was still statistically significant with low heterogeneity (RR: 1.82; 95% CI 1.49 to 2.23; *n* = 324; four studies; *I* ^2^ = 19%) (Figure 5).

#### 3.4.4. Recurrent Angina

**Figure 6:**
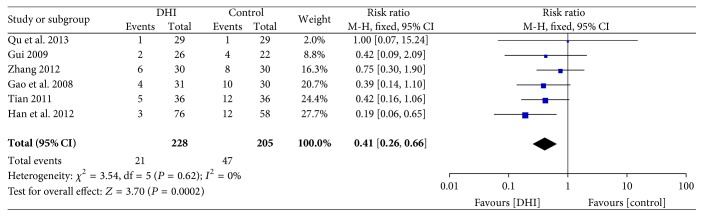
Forrest plot of recurrent angina.

Six studies [24, 25, 29, 32, 33, 36] assessed recurrent angina. Mete-analysis showed that compared with conventional treatment, conventional treatment plus DHI was associated with a statistically significant decrease in the risk of recurrent angina (RR: 0.41; 95% CI 0.26 to 0.66; *n* = 433; *I* ^2^ = 0%) (Figure 6).

#### 3.4.5. Arrhythmia

**Figure 7:**
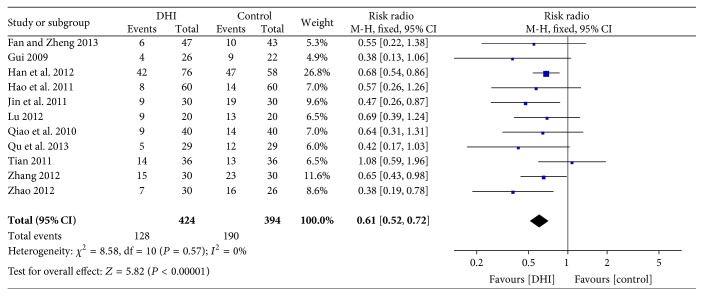
Forrest plot of arrhythmia.

**Figure 8:**
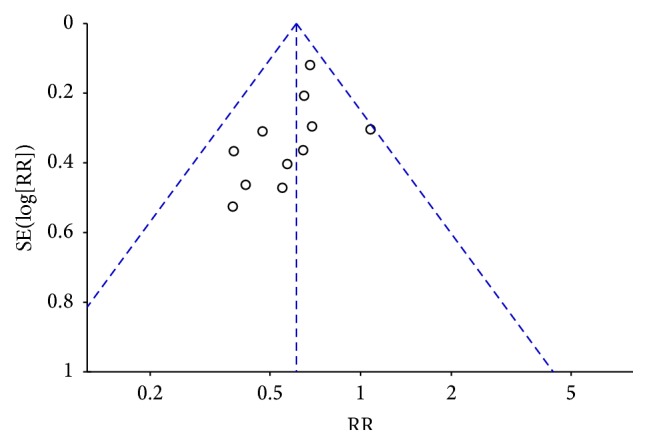
Funnel plot of arrhythmia.

Eleven trials [25, 26, 28–36] assessed participants who suffered from arrhythmia. The meta-analysis result manifested that conventional treatment plus DHI was associated with a statistically significant decline in the risk of arrhythmia compared with conventional treatment (RR: 0.61; 95% CI 0.52 to 0.72; *n* = 818; *I* ^2^ = 0%) (Figure 7). A funnel plot was applied to investigate the publication bias among these studies. The asymmetry of the funnel plot indicated that potential publication bias among studies might influence the result (Figure 8).

#### 3.4.6. Heart Failure

**Figure 9:**
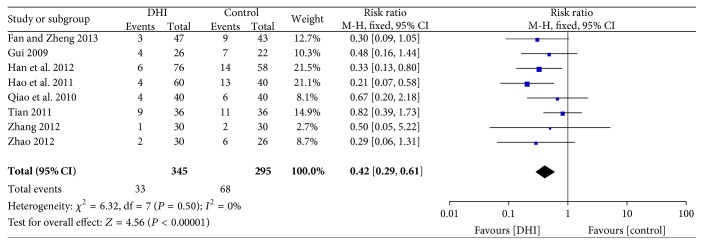
Forrest plot of heart failure.

Eight studies reported heart failure [25, 26, 29–33, 35]. Meta-analysis of these studies revealed that conventional treatment plus DHI was associated with a statistically significant reduction in risk of heart failure (RR: 0.42; 95% CI 0.29 to 0.61; *n* = 640; *I* ^2^ = 0%) (Figure 9).

#### 3.4.7. LVEF

**Figure 10:**
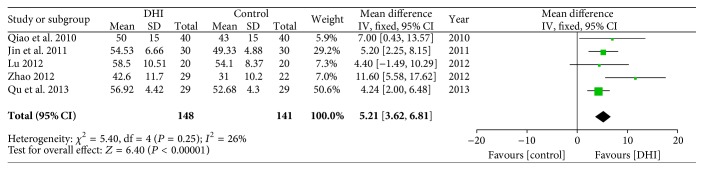
Forrest plot of left ventricular ejection fraction (LVEF).

Five studies reported the outcomes of LVEF [26, 28, 31, 34, 36]. Meta-analysis showed that conventional treatment plus DHI was associated with a statistically significant increase in LVEF compared with conventional treatment (MD: 5.21; 95% CI 3.62 to 6.81; *n* = 289; *I* ^2^ = 26%) (Figure 10).

#### 3.4.8. Adverse Events

**Figure 11:**
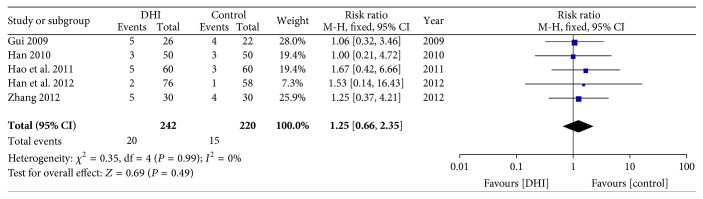
Forrest plot of bleeding events.

Six of the thirteen studies reported adverse events, while thirteen trials did not provide any related information [25, 27, 30, 32–34]. Among six studies, one trial [34] gave a narrative description of liver and kidney functions, showing no statistically significant difference between two groups. Other five studies [25, 27, 30, 32, 33] provided numerical cases of bleeding events in both groups. Therefore, a meta-analysis was performed on bleeding events, which found that DHI did not increase the risk of bleeding (RR: 1.25 95% CI 0.66 to 2.35; *n* = 462; *I* ^2^ = 0%) (Figure 11).

## 4. Discussion

Western medicine, whose aim is to restore blood flow to the ischemic myocardium, has made significant progress in the treatment of AMI with revascularization in past few decades and has tremendously reduced the death risk in AMI patients. However, the recent PEACE study found that the application of western medicine and modern technology such as PCI did not improve the mortality in China in the past decade [38]. Moreover, current studies have found that reperfusion to the ischemic myocardium can paradoxically reduce the beneficial effects of restored blood flow and even aggravate the necrosis of ischemic myocardium, leading to severe complications [39]. Chinese herbal medicinals have been widely applied in the treatment for disorders related to AMI in China since thousands of years ago and were regarded as natural products with better efficacy and less side effects. In china, DHI was one of the most widely used traditional Chinese herbal medicinals for AMI. Several researches implicated that DHI could inhibit the platelet activation and aggregation [40–42], which both play an important role in the process of acute myocardial infarction [43–45]. Recent studies have also found that DHI could protect ischemic myocardium against myocardial ischemia/reperfusion injury [46–48]. Plenty of clinical studies have reported the efficacy of DHI for AMI patients. Based on the previous evidence, a hypothesis was proposed that DHI might be a potentially effective drug in treating AMI patients. However, the efficacy and safety of DHI in treating AMI patients have not yet been critically evaluated. So systematical assessment of the efficacy and safety of DHI for AMI is significantly urgent and necessary.

In this systematic review, thirteen studies were included with a total of 979 participants. There was no statistically significant effect of conventional treatment plus DHI on recurrent AMI. However, conventional treatment plus DHI demonstrated statistically significant benefit in terms of mortality, reperfusion rate, arrhythmia, recurrent angina, heart failure, and improved LVEF as compared with conventional treatment.

In this review, DHI was found with no effect to increase the bleeding risk. However, due to the low quality of the included trials and inadequate data, we are unable to evaluate the safety of DHI for AMI patients at present. Hence, we appeal for a detailed description of adverse events in the future studies of DHI.

A number of limitations should be taken into consideration when accepting the findings of this review. Firstly, none of the thirteen included studies was assessed to be at low risk of bias. Thirteen trials claimed to have performed randomization, but only one [36] trial reported how their random sequence was generated and the rest did not. No study gave any information about allocation concealment. Thus, whether the randomization was effectively conducted in these trials was doubtful, which might lead to potential selection bias. Two studies [25, 33] were single-blinded while the others did not report blinding of participants or personnel. In all studies, the blinding of outcome assessment remained unknown. Insufficient reporting of blinding on participants, personnel, and outcome assessors might lead to potential performance bias and detection bias. Dropout account, withdrawals, and ITT analysis were not reported in any study, which might lead to potential attrition bias. Three studies [26, 27, 32] had high risk at selective reporting, which might lead to potential reporting bias. In addition, none of the included studies has reported the sample size estimation and most of the durations of follow-up were short, which weakened the validity of statistical analysis. Since the low quality of the included studies, the widespread popularity of traditional Chinese medicines, and the preference on traditional Chinese medicines of Chinese patients, it is hard to distinguish whether the effect of DHI was confounded by other traditional Chinese medicines. Therefore, we cannot draw a convincing conclusion that there were significant beneficial effects of DHI combined with conventional treatment compared with conventional treatment alone. Secondly, although extensive research was performed in several databases without setting any language limitation, all of the included studies in this review were published in Chinese, which lead to potential location bias. Thus, the asymmetry of the funnel plot indicated that the publication bias could not be excluded. Hence, reporting bias might exist in this review and exaggerate the results. Thirdly, except one study [33], the rest included studies failed to provide a detailed descriptions of their conventional treatment. Differences of conventional treatment among included studies contain potential confounding factors, which might influence the results of meta-analysis and reduce the validity of this review (e.g., angiotensin converting enzyme inhibitor was used in one trial as conventional treatment, while others did not. Meta-analysis might show a statistical difference. Statistical difference may not be caused by intervention but by confounding factor (ACEIs)). Fourthly, previous studies have demonstrated that physical activity and exercise appear to be beneficial for AMI patients [49–51]. Tai Chi, or Tai Chi Chuan, which was an exercise that originated from ancient Chinese martial arts and traditional Chinese medical theory, was also validated to have positive effect on patients with different diseases [52–54], including AMI patients [55, 56]. Since Tai Chi was widely popular among Chinese patients and it was easy to perform. However, in high quality and rigorous RCTs, it was intractable to detect it or to control it, not to mention in our poor quality included studies. Therefore, exercise like Tai Chi might be a potential confounder that might have an impact on our study results.

## 5. Conclusion

This systematic review finds potential benefits of DHI on AMI patients in terms of the incidence of mortality, reperfusion rate, arrhythmia, recurrent angina, heart failure, and LVEF, as compared with conventional treatment. However, the benefits should be cautiously considered due to the poor quality of evidence. In addition, the safety of DHI has not yet been verified for the deficiency of available studies. More high quality evidence from high quality RCTs is needed to support the clinical use of DHI for AMI patients.

## Figures and Tables

**Table 1:** Characteristics of included studies.

ID	Diagnostic criteria	Type of AMI	Sample size (I/C)	Baseline	Duration of treatment	Follow-up	Experiment group	Control group	Outcomes
Gao et al. 2008 [24]	2004 ACC/AHA	STEMI	61 (31/30)	Yes	2 weeks	2 weeks	CT + DHI 40 mL.qd.ivd.gtt	CT (+PCI)	Recurrent angina, ECG, and aPTT

Gui 2009 [25]	Not specific	Unclear	48 (26/22)	Yes	2 weeks	2 weeks	CT + DHI 20 mL.qd.ivd.gtt	CT (+thrombolysis)	Mortality, recurrent AMI, shock, HF, arrhythmia, rehospitalization, recurrent angina, and adverse events

Qiao et al. 2010 [26]	Not specific	Unclear	80 (40/40)	Yes (narrative only)	2–4 weeks	2–4 weeks	CT + DHI 40 mL.qd.ivd.gtt	CT	Arrhythmia, HF, shock, reperfusion, and LEVF

Han 2010 [27]	Not specific	Unclear	100 (50/50)	Yes (narrative only)	4 weeks	4 weeks	CT + DHI 20 mL.qd.ivd.gtt	CT (+thrombolysis)	Mortality, adverse events, and reperfusion rate

Jin et al. 2011 [28]	Not specific	Unclear	60 (30/30)	Yes	2 weeks	2 weeks	CT + DHI 30 mL.qd.ivd.gtt	CT (+thrombolysis)	Myocardial enzyme, arrhythmia, LEVF, WMSI, ECG, t-PA, PAI-1, CRP, and Fib

Tian 2011 [29]	WHO	STEMI	72 (36/36)	Yes (narrative only)	2 weeks	2 weeks	CT + DHI 30 mL.qd.ivd.gtt	CT	Recurrent angina, HF, arrhythmia, ECG, and BP

Hao and Ren 2011 [30]	2001 CSCCMA	Unclear	120 (60/60)	Yes (narrative only)	Not specific	Not specific	CT + DHI 30 mL.qd.ivd.gtt	CT (+thrombolysis)	Mortality, shock, HF, reperfusion, arrhythmia, and adverse events

Zhao 2012 [31]	Not specific	Unclear	56 (30/26)	Yes	2 weeks	2 weeks/3 months	CT + DHI 30 mL.qd.ivd.gtt	CT (+thrombolysis)	Mortality, myocardial enzyme, HF, arrhythmia, recurrent AMI, LEVF, rehospitalization, and reperfusion

Han et al. 2012 [32]	2001 CSCCMA	Unclear	134 (76/58)	Yes (narrative only)	2 weeks	4 weeks	CT + DH 30 mL.qd.ivd.gtt	CT (+thrombolysis)	ECG, arrhythmia, myocardial enzyme, reperfusion, HF, shock, recurrent angina, mortality, and adverse events

Zhang 2012 [33]	2001 CSCCMA	Unclear	60 (30/30)	Yes	1 week	1 week	CT + DHI 30 mL.qd.ivd.gtt	CT (+thrombolysis)	Arrhythmia, recurrent angina, HF, myocardial enzyme, hs-CRP, NT-pro-BNP, and adverse events

Lu 2012 [34]	2009 CSCCMA	NSTEMI	40 (20/20)	Yes	7–10 days	7–10 days	CT + DHI 20 mL.qd.ivd.gtt	CT (+PCI)	IL-6, NO, ET, arrhythmia, LEVF, myocardial enzyme, and adverse events

Fan and Zheng 2013 [35]	Not specific	Unclear	90 (47/43)	Yes (narrative only)	Not specific	Not specific	CT + DHI (without specific usage)	CT (+thrombolysis)	Mortality, HF, shock, reperfusion, and arrhythmia

Qu et al. 2013 [36]	2010 CSCCMA	Unclear	58 (29/29)	Yes	2 weeks	2 weeks	CT + DHI 40 mL.qd.ivd.gtt	CT (+thrombolysis)	Arrhythmia, recurrent angina, myocardial enzyme, reperfusion, WMSI, and LEVF

Notes: AHA: American Heart Association; ACC: American College of Cardiology; WHO: World Health Organization; CT: conventional therapy; DHI: Danhong injection; ECG: electrocardiography; CSCCMA: Chinese Society of Cardiology of Chinese Medical Association; recurrent AMI: recurrent acute myocardial infarction; STEMI: ST-segment elevation myocardial infarction; NSTEMI: non-st-segment elevation myocardial infarction; HF: heart failure; LEVF: left ventricular ejection fraction; WMSI: wall motion score index; NO: Nitric Oxide; ET: endothelin; t-PA: tissue-type plasminogen activator; PAI-1: Plasminogen Activator Inhibitor 1; CRP: C-reaction protein; hs-CRP: high-sensitivity CRP; Fib: fibrinogen; NT-pro-BNP: n-terminal probrain natriuretic peptide; IL-6: interleukin-6; PCI: percutaneous coronary intervention.
